# Electrochemical and electrophysiological considerations for clinical high channel count neural interfaces

**DOI:** 10.1557/s43577-023-00537-0

**Published:** 2023-05-31

**Authors:** Ritwik Vatsyayan, Jihwan Lee, Andrew M. Bourhis, Youngbin Tchoe, Daniel R. Cleary, Karen J. Tonsfeldt, Keundong Lee, Rhea Montgomery-Walsh, Angelique C. Paulk, Hoi Sang U, Sydney S. Cash, Shadi A. Dayeh

**Affiliations:** Integrated Electronics and Biointerfaces Laboratory, Department of Electrical and Computer Engineering, University of California, San Diego, San Diego, USA; Integrated Electronics and Biointerfaces Laboratory, Department of Electrical and Computer Engineering, University of California, San Diego, San Diego, USA; Integrated Electronics and Biointerfaces Laboratory, Department of Electrical and Computer Engineering, University of California, San Diego, San Diego, USA; Integrated Electronics and Biointerfaces Laboratory, Department of Electrical and Computer Engineering, University of California, San Diego, San Diego, USA; Integrated Electronics and Biointerfaces Laboratory, Department of Electrical and Computer Engineering, University of California, San Diego, San Diego, USA; Department of Neurological Surgery, School of Medicine, Oregon Health & Science University, Portland, USA; Integrated Electronics and Biointerfaces Laboratory, Department of Electrical and Computer Engineering, University of California, San Diego, San Diego, USA; Department of Obstetrics, Gynecology, and Reproductive Sciences, Center for Reproductive Science and Medicine, University of California, San Diego, San Diego, USA; Integrated Electronics and Biointerfaces Laboratory, Department of Electrical and Computer Engineering, University of California, San Diego, San Diego, USA; Integrated Electronics and Biointerfaces Laboratory, Department of Electrical and Computer Engineering, University of California, San Diego, San Diego, USA; Department of Bioengineering, University of California, San Diego, San Diego, USA; Department of Neurology, Harvard Medical School, Boston, USA; Center for Neurotechnology and Neurorecovery, Department of Neurology, Massachusetts General Hospital, Boston, USA; Integrated Electronics and Biointerfaces Laboratory, Department of Electrical and Computer Engineering, University of California, San Diego, San Diego, USA; Department of Neurology, Harvard Medical School, Boston, USA; Center for Neurotechnology and Neurorecovery, Department of Neurology, Massachusetts General Hospital, Boston, USA; Integrated Electronics and Biointerfaces Laboratory, Department of Electrical and Computer Engineering, University of California, San Diego, San Diego, USA; Department of Bioengineering, University of California, San Diego, San Diego, USA

## Abstract

Electrophysiological recording and stimulation are the gold standard for functional mapping during surgical and therapeutic interventions as well as capturing cellular activity in the intact human brain. A critical component probing human brain activity is the interface material at the electrode contact that electrochemically transduces brain signals to and from free charge carriers in the measurement system. Here, we summarize state-of-the-art electrode array systems in the context of translation for use in recording and stimulating human brain activity. We leverage parametric studies with multiple electrode materials to shed light on the varied levels of suitability to enable high signal-to-noise electrophysiological recordings as well as safe electrophysiological stimulation delivery. We discuss the effects of electrode scaling for recording and stimulation in pursuit of high spatial resolution, channel count electrode interfaces, delineating the electrode–tissue circuit components that dictate the electrode performance. Finally, we summarize recent efforts in the connectorization and packaging for high channel count electrode arrays and provide a brief account of efforts toward wireless neuronal monitoring systems.

## Introduction

Understanding the ongoing dynamics of neural activity in the human brain—how these dynamics support function, and how brain activity relates to pathologies—remain one of the big-gest challenges of the 21st century. Such an understanding can serve the development of treatment paradigms for debilitating neurological disorders, including Alzheimer’s disease, epilepsy, multiple sclerosis, paralysis, and traumatic brain injury.^1,2^ In the United States alone, there are more than 53 million people living with a neurological pathology, and its impact on the economy is colossal, exceeding US$1.5 trillion annually.^3,4^ Recent multidisciplinary progress has allowed us to attain an informative yet limited understanding of how function and disease are presented by the tens of billions of neurons in the human brain.^5^ Critical to our progress in understanding human brain activity are the technological advances that resolve and modulate neurophysiological activity, ideally at the cellular level though, at this point, most of the research has involved recording from thousands of neurons in large areas of the brain.^6,7^ However, we are now at an inflection point where technological advances in electrodes could fill significant gaps in our spatial and temporal sampling of human brain activity.

Of course, there are questions on whether high spatial resolution brain activity sampled with high signal-to-noise ratio is needed in recording human brain activity, whether to answer neuroscientific questions, or if it has clinical uses. Indeed, many clinical indications, to date, are dependent on coarse spatial resolution recording and stimulation electrodes. However, we and other groups have been building increasing evidence that high-resolution brain recordings at the single cell or local population level could inform us as to the neural mechanisms underlying functional and pathological brain activity, including epilepsy, cognition, and enable motor control for brain–computer interfaces.^8–15^ Therefore, advances in electrode technologies that provide increased high-resolution activity could be key for addressing fundamental neuroscientific problems as well as answering questions around pathology that current clinical techniques do not afford.

**Figure 1.**
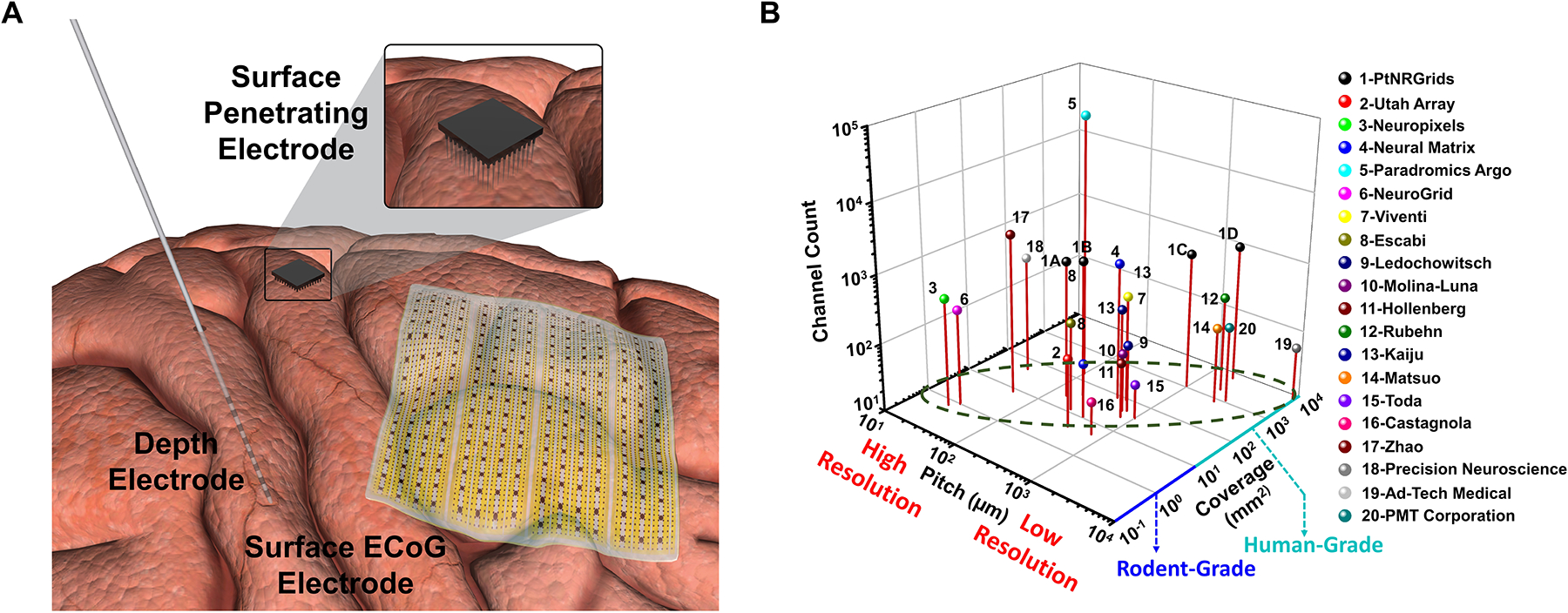
Comparisons between different types of electrodes and the Resolution-Coverage tradeoff. (a) Representative positioning of the different electrode types (surface electrocorticography [ECoG], depth, and penetrating surface) on the surface of the brain (illustrations not to scale). (b) Comparison of the inter-contact pitch, total coverage, and channel count offered by the state-of-the-art recording electrodes: 1-PtNRGrids,^33^ 2-Utah Array,^31^ 3-Neuropixels,^32^ 4-Neural Matrix,^42^ 5-Paradromics Argo,^36,43^ 6-NeuroGrid,^44^ 7-Viventi,^45^ 8-Escabi,^46^ 9-Ledochowitsch,^47^ 10-Molina-Luna,^48^ 11-Hollenberg,^49^ 12-Rubehn,^50^ 13-Kaiju,^51^ 14-Matsuo,^52^ 15-Toda,^53^ 16-Castagnola,^54^ 17-Zhao,^55^ 18- Precision Neuroscience,^56^ 19-Ad-Tech Medical Clinical Grid, 20-PMT Corporation Clinical Grid.^57^ The dashed region shows the tradeoff between the channel pitch and coverage for devices with limited channel count.

Currently, brain activity is sampled with noninvasive and invasive tools. Noninvasive imaging techniques such as functional magnetic resonance imaging (fMRI), scalp electroen-cephalography (EEG), and magnetoencephalography (MEG) provide coarse temporal and spatial resolution.^16^ Invasive electrophysiological techniques using stereo-encephalography (sEEG) and electrocorticography (ECoG) electrodes provide better temporal resolution, and can have high, local spatial resolution or broad brain coverage (Figure 1). Further, for some of these devices, direct electrical stimulation can be delivered to examine function as well as pathology. sEEG (depth) and ECoG (surface) electrodes are the current standard of clinical care in the diagnosis to inform the treatment of epilepsy and brain tumors that involves precise surgical procedures and implantation of therapeutic devices.^17,18^ Yet a problem is that the electrodes, or devices, currently used in clinical sampling use materials discovered and used for the past 50 years, having primary technical limitations residing at a fundamental materials level. The focus of this article is on the electrochemical and electrophysiological considerations needed to be addressed to attain high spatial resolution, widespread, and high channel count electrode arrays.

Neuroimplantable devices can be classified according to the duration of their use as acute (<24 h), semi-chronic (≤30 days), and chronic (>30 days), and the target location of surface or depth of the brain (Figure 1a). Acute devices are typically one-time use within the operating room (intraoperative) for the mapping of healthy and diseased brain activity (e.g., epileptiform activity). Semi-chronic devices are typically used for diagnostic purposes in a controlled hospital environment, such as the epilepsy monitoring unit (EMU).^19^ Some chronically implanted devices can be used to deliver targeted therapies to stimulate and record ongoing activity for specific neurodegenerative conditions such as Parkinson’s disease and epilepsy,^20,21^ and deliver high-frequency stimulation through epidural spinal-cord stimulation to manage intractable pain.^22^

In addition, implantable electrodes can be classified based on whether they are inserted into the brain parenchyma or on the surface. Surface ECoG arrays are typically placed on the surface of the brain while sEEG or DBS electrodes penetrate the brain tissue to record from neural populations in deep brain structures such as the hippocampus, thalamus, and substantia nigra, for the control of seizures, deep brain stimulation for Parkinson’s disease, depression, and intractable epilepsy.^23–25^ There are also surface penetrating electrodes, such as the Utah array and nanowire arrays, which provide access to superficial layers of the cortex and can record single and multiunit activity in a 4 × 4 mm^2^ area, laminar arrays^26,27^ and Neuropixels probes^28,29^ with access to activity along cortical layers at high spatial resolution. Finally, a recent advance has been the use of stentrodes to record brain activity chronically from within blood vessels in the brain.^30^

The current state-of-the-art technology for surface and depth electrodes illustrates the stark contrast between clinically approved devices and cutting-edge technologies, particularly the tradeoff between the inter-contact pitch, spatial coverage, and channel count provided by the state-of-the-art electrode technologies (Table I; Figure 1b). In general, the tradeoff is that higher spatial resolution devices (even those that can be mass-produced) usually cover a smaller surface area of the brain whereas current clinical electrodes cover larger areas of the brain but are coarse (>4 mm) spatial resolution and require extensive hand assembly. In addition, decreasing these metal electrode contact sizes to achieve higher spatial resolution results in added 1/f and thermal noise that compromise the recording fidelity. Yet, high spatial resolution electrodes such as the Utah array and the Neuropixels probes can reach high channel counts up to a few thousand contacts for a relatively small coverage area (Figure 1b).^31,32^ To mitigate this tradeoff, new fabrication and interconnection strategies evolved to achieve thousands^33–35^ to tens of thousands^36^ of electrode channels in a variety of species, including humans.^37^

Other limitations to achieving high channel count neural interface systems include expanding components to acquire, display, and interpret high-dimensional neuronal activity. For example, acquisition electronics are required to record and amplify neuronal activity transduced by the electrode while simultaneously rejecting noise and external disturbances.^38^ These systems must operate on a low-power budget to ensure the safety of the patient, as well as to overcome the limitations in charge-storage and power-telemetry technology. Further, to be eligible for chronic applications, these devices must be hermetically sealed to ensure high-fidelity performance over time in the presence of biological agents such as blood and cerebrospinal fluid. The software used to visualize neuronal data must handle and display large data sets at high data rates (e.g., 1.96 Gbps for 4096 channels at 30 kHz sampling frequency and 16-bit resolution) where automated algorithms and dimensionality-reduction techniques become necessary to facilitate real-time decision-making for surgical or closed-loop applications.^39^ These other limitations in the back end of the electrode devices are summarized in other review articles.^40,41^ Here, we will primarily focus our discussions on the first stage of data acquisition: electrode technology to record and stimulate the neuronal activity.

## Recording interfaces

To achieve high density and high coverage recording interfaces, the electrode contact dimensions need to scale without substantially increasing its electrochemical impedance. The high impedance causes higher 1/f and thermal noise.^64^ Further, a high electrode impedance and a high parasitic capacitance for metal traces can attenuate the amplitude of the recorded signal.^65^ Further, contacts with impedances com-parable to the parasitic capacitance between the metal leads will be more susceptible to crosstalk between channels.^33,66^ Thus, metal leads need to be kept as short as is possible, and the electrode contact needs to have as low an impedance as possible.

**Figure 2.**
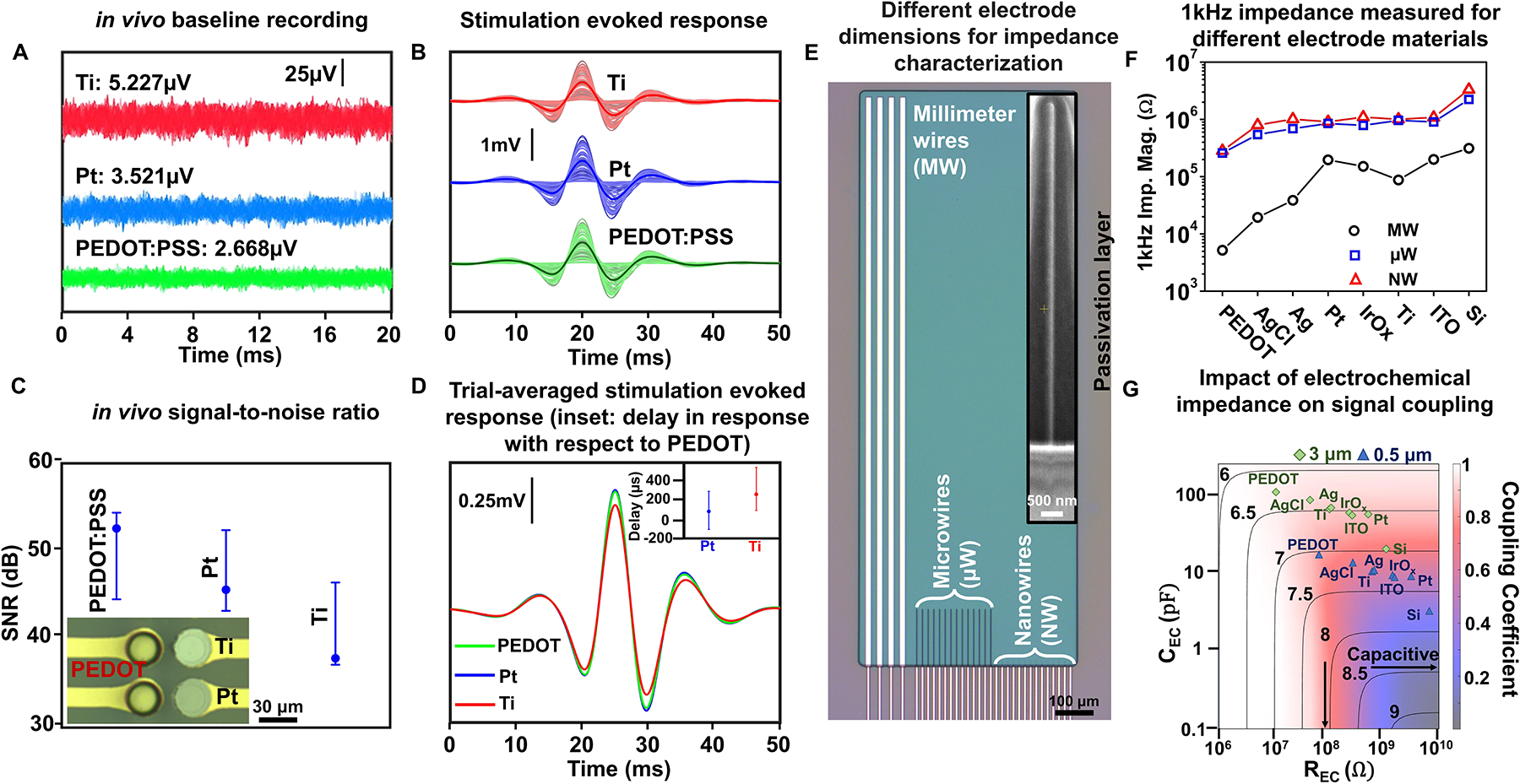
Electrode material differences in impedance impact how the neural signal is recorded. (a–d) Comparison of recorded data from 30-μm diameter Ti, Pt, and PEDOT:PSS electrode contacts, sampled at 20 kHz. The three contacts are placed spatially adjacent to each other, as shown in the inset of panel (c). (a) Baseline noise recorded from Ti, Pt, and PEDOT:PSS electrode contacts *in vivo*, high-pass filtered at 300 Hz, and the corresponding root mean square (RMS) noise recorded on each material for a 20-ms recording. (b) Filtered high-gamma activity (70–190 Hz) recorded *in vivo* on the barrel cortex of a rat in response to an air puff stimulation applied on the whisker. The data show the response plotted for multiple trials, with the average trial-averaged waveform plotted in bold for each material. (c) The corresponding signal-to-noise ratio (SNR) of the three materials extracted from the results plotted in (a) and (b). (d) The trial-averaged response measured on each material, comparing the relative variation in the maximum amplitude measured on each material, aligned to the onset of air stimulation. The inset shows the average delay in the positive peak of the response on the Pt (89.5 μs) and Ti (268 μs) contacts, with respect to the PEDOT contact. (e) Optical and electron microscope images of nanowire, microwire, and millimeter wire electrodes used to study the variation of the (f) 1 kHz impedance, in benchtop measurements, for different electrode contact materials. (g) The variation of the coupling coefficient as a function of the equivalent R and C at the electrochemical interface.^67^

To illustrate the impact of the electrode impedance on the recording performance, we directly compared the performance of three contact materials, titanium (Ti, 1.5 MΩ at 1 kHz), planar platinum (Pt, 400 kΩ at 1 kHz), and poly(3,4-ethylene-dioxythiophene) poly(styrene sulfonate) (PEDOT:PSS, 30 kΩ at 1 kHz) with 30-μm contact diameter and 50-μm center-to-center spacing (Figure 2c) in the same electrode array to essentially record the same neurophysiological activity and perform the same baseline recordings using different material contacts. These contacts were placed on the sensory whisker barrel cortex of rats, which is responsive to mechanical deflection of the contralateral whiskers. The location of the barrel cortex on the anesthetized rat’s brain was predetermined using a high-density 1024 channel PtNR ECoG array using established procedures in our laboratory.^33^ We first recorded the baseline activity over the barrel cortex without any external stimuli and high-pass filtered the recordings above 300 Hz to remove the effect of local field potentials (LFPs) (Figure 2a). The variance in the signal increases with electrode impedance and is highest for the Ti contact, and lowest for the PEDOT:PSS contact, as indicated by the RMS of the signal recorded on each contact. With whisker deflections, the response for the PEDOT:PSS and Pt contacts were essentially similar, whereas that of the Ti contact was smaller, as shown in Figure 2d. For the Ti contact, the impedance becomes nearly 10% of the input amplifier impedance value (16 MΩ at 1 kHz). Additionally, we also observed a delay in the measured response peak across different materials, with the response peak on the Ti contact occurring on an average 268 μs after the peak on the PEDOT:PSS contact. Although typical neural signal propagation speed can be 0.2–1 m/s, the inter-contact separation between the PEDOT:PSS, Pt, and Ti contacts was 50 μm leading to a worst-case delay of 50 μs. The delay observed on the Ti contact is significantly higher, which we associate with the charging delay across the electrode-amplifier interface^67^ (Figure 2c).

To further test this, we simulated the effects of extreme scaling of electrode contacts across millimeter, micrometer, and nanometer scales (Figure 2e–f).^67,68^ For the simulations, we define the coupling coefficient as the ratio of the recorded signal amplitude to the input signal amplitude at the electrode surface (Figure 2g). Though the magnitude of the impedance at 1 kHz remains essentially the same at the micrometer and nanometer scales due to the dominance of edge effects, we observed a pronounced impact of the capacitive and resistive parts of the electrochemical impedance on the coupling coefficient (Figure 2g). These results further illustrate that the material surface properties can still have profound impact on the recording fidelity at deeply scaled contact dimensions.

**Figure 3.**
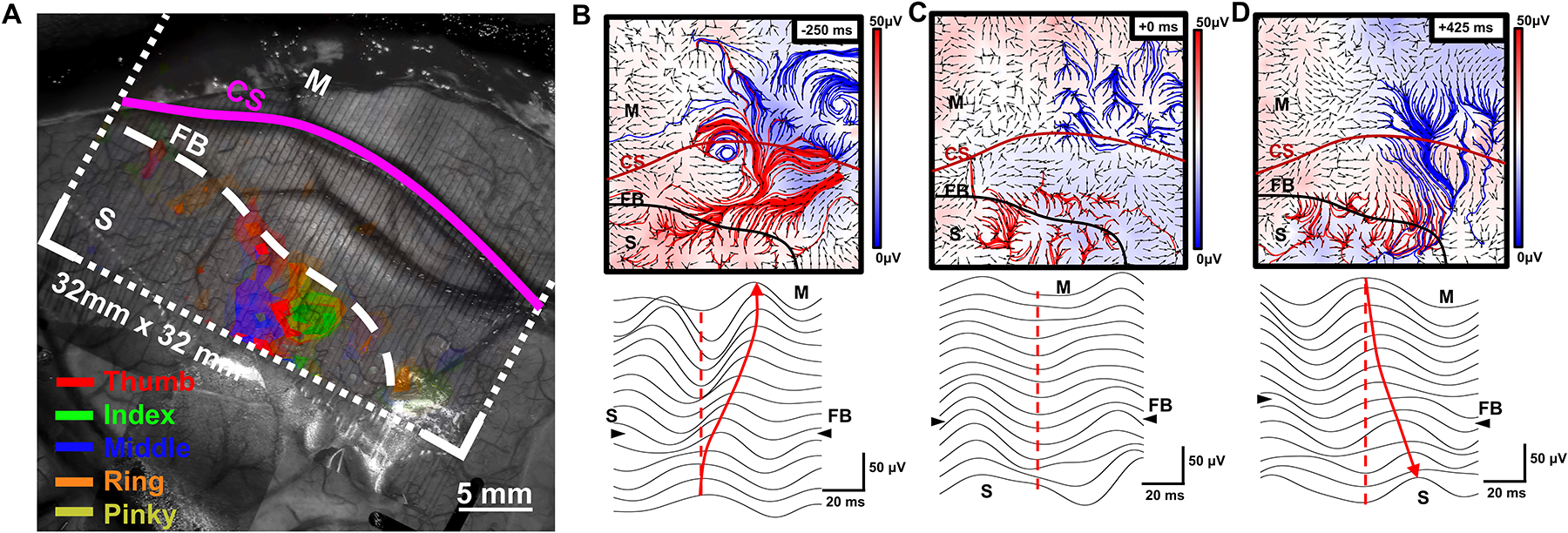
Recorded activity using a high-density, high channel count PtNRGrid placed on the surface of a human brain during a craniotomy. (a) Overlay plot of high-gamma activity sensory responses superimposed on top of a photo of the surface of a patient’s brain, in response to vibrotactile stimulation of individual fingers of the patient. Functional boundary (FB). (b–d) The patient is asked to perform a grasping task using the hand, and the measured propagating beta waves and waveforms are plotted across the central sulcus (CS) in the (b) planning stage of the motion, (c) during the motion, and (d) after the completion of motion of a patient’s hand. The red and blue streamlines originate from the sensory (S) and motor (M) cortices, respectively. The background color represents the amplitude of the beta wave potential, and the arrowheads indicate the propagating direction of the beta waves. Bottom plots are raw waveforms around the time stamps of (b) to (d), with the arrowheads indicating the propagation direction of the beta waves.^33^

The potential for the use of high-density, high channel count arrays in recording neuronal activity across a large area of cortex with unprecedented detail has recently been illustrated.^33^ PtNRGrids with 1024 channels resolved the curvilinear nature of the functional boundary (FB) between the somatosensory (S1) and somatomotor (M1) regions and localized individual finger units by vibrotactile stimulation and high-gamma mapping within the identified S1 region (Figure 3a). Additionally, PtNRGrids resolved novel large-scale spatiotemporal dynamics as the subject performed a hand grasping motion (Figure 3b–d). The high-density low noise recording of high precision mapping of the brain neurodynamics hold the potential to reveal the neuronal underpinnings of normal and diseased brain function.

## Stimulating interfaces

The pulsed direct electrical stimulation in the brain, the spine, and the peripheral nervous system has long been used clinically for both diagnostic and therapeutic applications.^69,70^ Electrophysiological stimulation is used acutely for neuromonitoring and functional mapping in common surgical procedures^71^ and chronically to treat neurodegenerative disorders such as Parkinson’s disease and Alzheimer’s disease.^72^ While noninvasive stimulation such as transcranial magnetic stimulation^73^ and transcranial direct current stimulation^74^ has been used for decades in cognitive neuroscience to modulate the neural activity in the brain, it provides more coarse spatial resolution neuromodulation than invasive direct electrical stimulation.

The impact of the electrode material and its electrochemical impedance is more pronounced in stimulation compared to recording. The typical metric used to compare stimulation interfaces is the charge-injection capacity (CIC) of the material, which is the total amount of charge per unit area of the stimulating contact that can be safely injected in tissue, without inducing damage in the tissue, or the stimulating interface itself.^75^ Typically, the amount of charge delivered into tissue is limited by the electrical potential that builds up on the stimulating interface, which in turn, is directly proportional to the electrode impedance. Therefore, materials with a high geometrical surface area and low impedance materials are more suitable for stimulation.

Because stimulation involves the delivery of electrical charge into the tissue, there are significantly higher safety con-cerns associated with it compared to passive recording. Historically, Shannon’s Equation has been adopted to determine the tissue damage thresholds.^76^ Later studies determined empirical safety limits of 30 μC/cm^2^ for macro-contacts and 4 nC/ph for micro-contacts.^77,78^ Indeed, the variation of the safety limit with the media surrounding the electrode needs to be accounted for. The safety thresholds for *in vivo* stimulation in tissue has been determined to be significantly lower than that established from benchtop measurements, primarily due to current spread and its impact of the effective electrode-medium impedance.^79^

**Figure 4.**
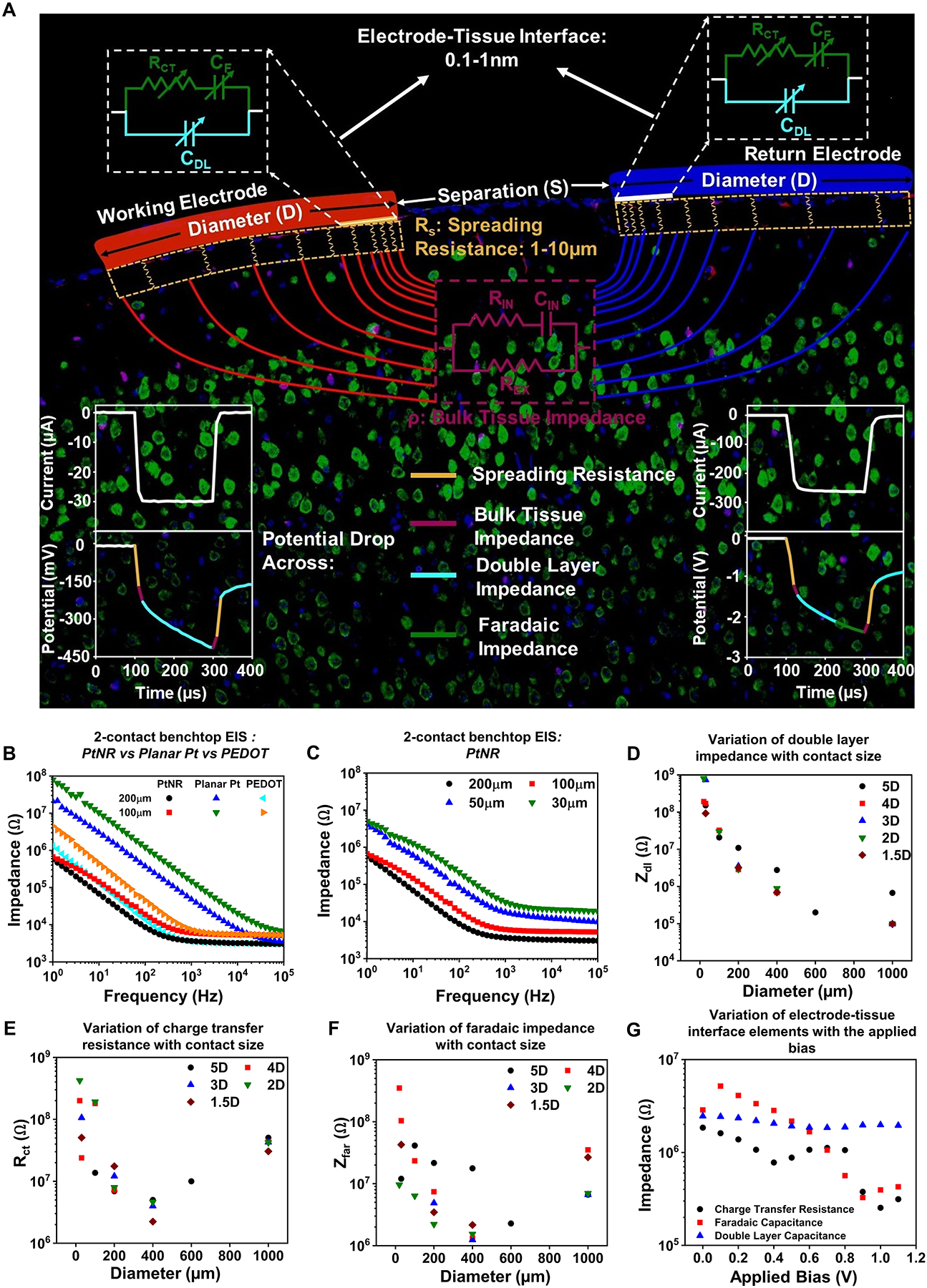
Direct electrical stimulation via intracranial electrodes to drive neural activity and the effects of electrode material and size on the measured impedance spectra. (a) The equivalent circuit model for current injection *in* vivo, showing the individual elements of the electrode–tissue interface that participate in the charge-injection process individually delineated. (b) Material- and (c) diameter-dependent electrical impedance spectra (EIS) in benchtop measurements. The diameter dependence of the (d) double layer impedance, (e) charge-transfer resistance, and (f) faradic impedance. (g) The bias-dependent variation of the electrochemical interface elements in benchtop.^80^

To understand the parameters that dictate safety in electrophysiological stimulation, we need to first understand the electrochemical interface, which participates in the charge-injection process. In a typical bipolar stimulation setup *in vivo*,^80^ the current is injected from the working electrode, and extracted from the return electrode, both of which have an identical contact diameter (D), and the separation (S) between the contacts can be varied (Figure 4a). The electrode–tissue interface consists of three main components: the capacitive network for charge injection formed by the double layer capacitance and the redox branch at the interface; the current crowding dictated spreading resistance within the tissue near the contact perimeter that faces the contact for current return; and the conduction impedance for current flow through the bulk tissue. The values for individual components of the interface elements can be calculated from the electrochemical impedance spectra (EIS) of the contact. In this setup, we show the magnitude and phase of the electrochemical impedance as a function of frequency for different materials at two diameters and for different diameters for the same material (Figure 4b–c). It is worth noting that this impedance is nonlinear and fractional, depending on the voltage across it and the ionic species present at any given time; thus, we must use nonlinear circuit elements, or make a line-arized approximation when fitting circuit elements to the EIS.^80^

The safety threshold for charge injection is determined by the potential buildup at the capacitive network at the interface. This network consists of a very thin layer of ions within 0.1–1 nm from the surface of the contact where the free charge carriers reside. At equilibrium, the capacitive charge screening element of interface is depicted with a constant-phase element, which describes a nonideal double-layer capacitor *C*_*DL*_, whose reactance *Z*_*DL*_ has a weaker dependence on frequency compared to ideal capacitors. Current at this interface can also be injected directly by charge transfer between the electrode and tissue. The direct charge injection is depicted by a resistive charge-transfer element *R*_*CT*_ and a constant-phase element *C*_*F*_ that captures the effect of ion migration to and from the double layer, although this motion is limited to the vicinity of the electrode and can participate in a redox reaction. The magnitudes of the interface elements vary with the size of the contact and with the contact spacing as is thoroughly discussed in Reference 80 (Figure 4d–f).

The difference in the Fermi energy level of the electrode contact and the electrochemical potential of the tissue governs the energy barrier for the charge-transfer process. When a bias is applied to inject current into the tissue (for voltage clamped stimulation), or when an injected current gives rise to a potential buildup at the interface (for current clamped stimulation), this energy barrier is overcome with direct charge transfer. The higher the applied bias, the more efficient this charge-transfer process is. Consequently, each element of the interface is bias-dependent because the stimulation process causes the electrode–tissue interface to deviate from equilibrium, the operational regime where EIS is typically conducted, as seen in the variation of the capacitive elements of the interface with the applied bias (Figure 4g).

**Figure 5.**
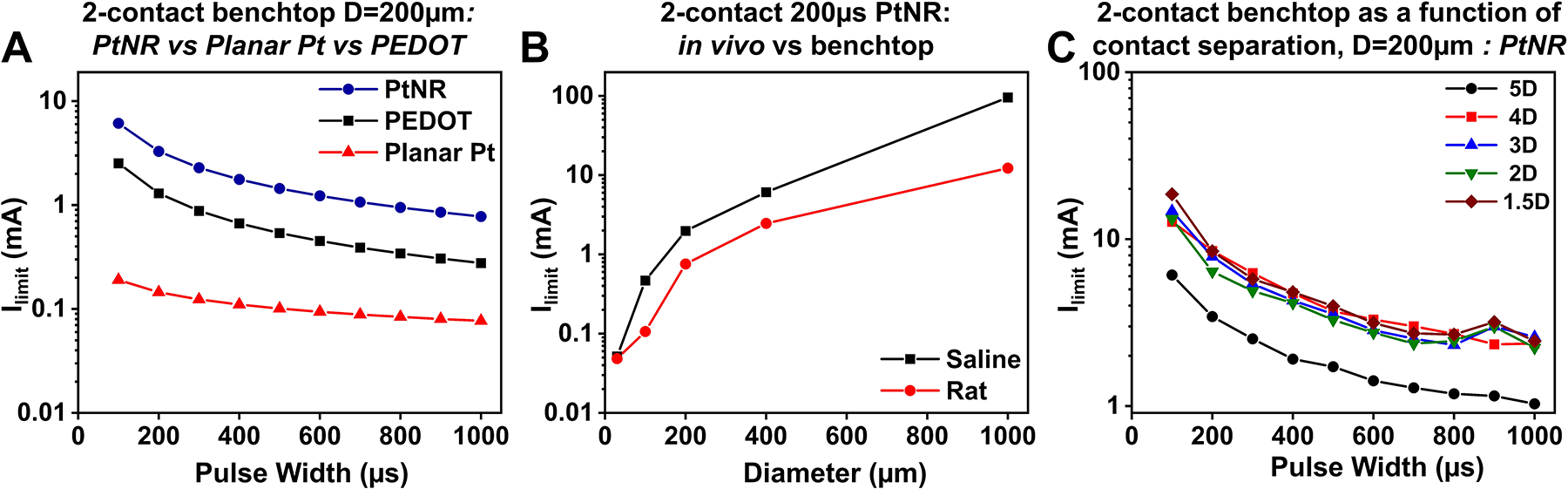
Dependence of maximum injectable current on stimulation design parameters. Maximum injectable current as a function of (a) pulse width for 200-μm PtNR, PEDOT:PSS, and planar Pt contacts, (b) diameter for a 200-μs pulse for *in vivo* (rat) and benchtop placement, (c) pulse width for a 200-μm PtNR contact for different inter-contact separations.^80^

Electrochemical damage at the stimulating electrode is caused by the excess buildup of potential that overcomes the energy barrier for irreversible reactions. Typically, the first irreversible reaction observed is the electrolysis of water.^81,82^ Cyclic voltammetry (CV) measurements, in which a voltage excursion is applied on the electrode and the resultant current is sampled, are typically carried out to establish the electrolysis window for the given material.^83^ To establish the CIC, a current-clamped square wave stimulation can be applied at the electrode, and the resulting excursion potential on the electrode can be measured. The current value for which this electrode excursion potential exceeds the electrolysis limit determined from the CV measurement is considered the current injection limit *I*_*limit*_ as shown in the dependence of *I*_*limit*_ with the electrode and stimulation design parameters for a bipolar stimulation setup (Figure 5a–c).

The electrode material primarily determines excursion potential and the current safety limit. Low impedance materials such as PtNR and PEDOT have significantly higher current safety thresholds compared to planar contact materials such as planar Pt (Figure 5a). As the pulse width of the injected current increases, *I*_*limit*_ decreases, as more charge is injected per phase. This change is nonlinear because longer current pulses allow more time for the interface to be modified, and the faradic elements of the charge-injection process tend to dominate more. *I*_*limit*_ also varies between benchtop (usually phosphate buffered saline) and *in vivo* (Figure 5b) because the interface impedance that depends on the surrounding media is higher *in vivo*. The inter-contact separation for bipolar stimulation also impacts *I*_*limit*_. For smaller inter-contact separations, the fringing fields are less pronounced, and the double layer is more efficient in charge injection. For larger separations, the location of the return contact becomes large enough to not impact the working contact, and the nonlinearities due to the fringing fields at the working contact tend to dominate thus lowering *I*_*limit*_ (Figure 5c).

**Figure 6.**
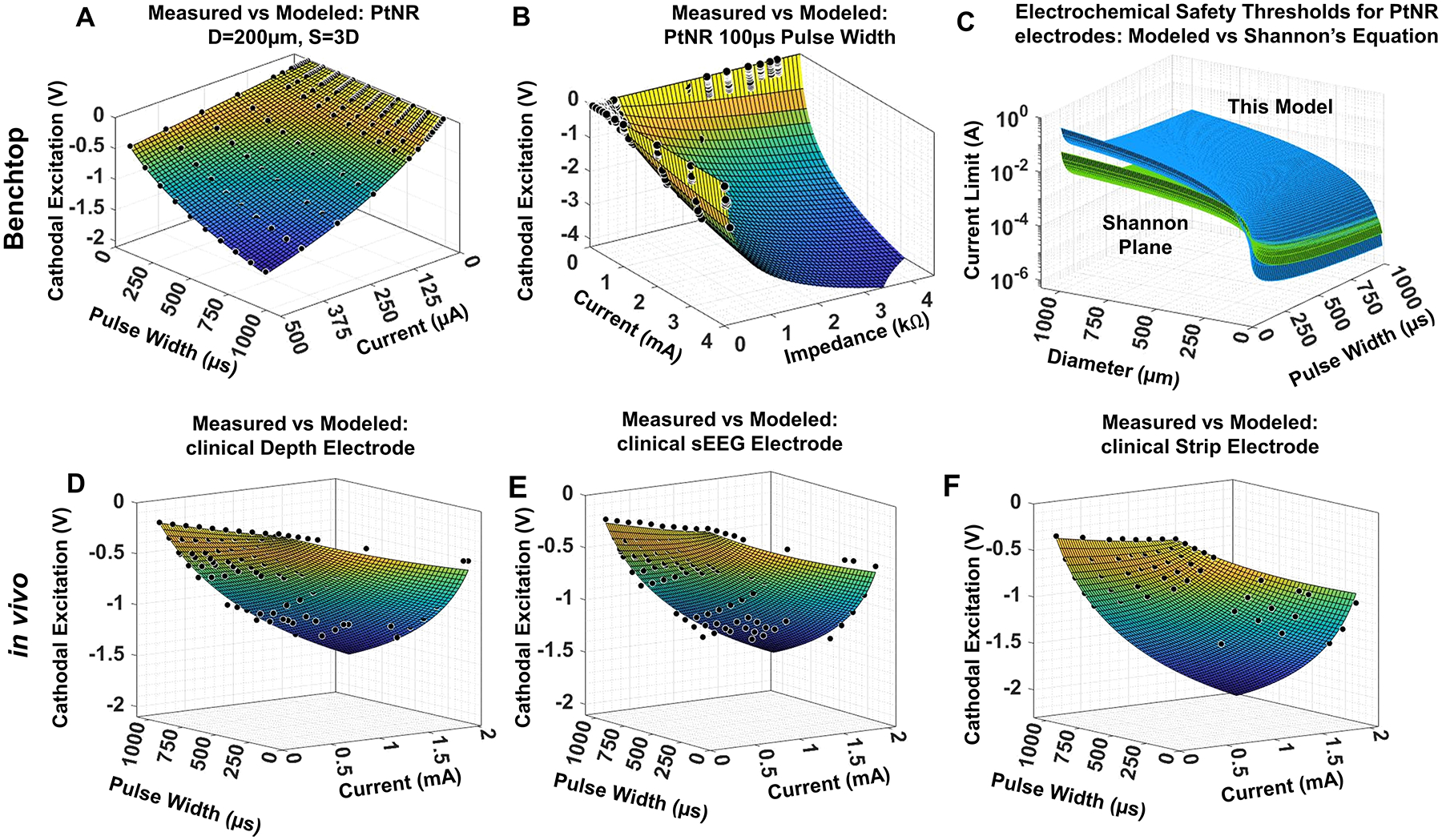
(a) The agreement between the modeled and simulated data for the cathodal excitation as a function of the pulse width and current. (b) The agreement between the modeled and simulated data for the cathodal excitation as a function of the current and impedance. (c) The safety limits predicted by the predictive equation compared to the limits proposed by Shannon’s Equation. (d–f) The fitting results for the cathodal excitation measured on the pig’s cortex using clinical electrodes, plotted as a function of the input current and pulse width, for (d) depth, (e) stereo-encephalography (sEEG), and (f) surface strip electrodes.^80^

Parametric studies such as those shown in Figures 4 and 5 allowed us to develop a more generalized equation predicting the electrochemical safety limits for stimulation.^80^ The potential built up on the electrode–tissue interface as a function of the injected current, pulse width and the electrochemical impedance can be expressed as

1 $$V_{\text{elec}}=a\left[ln\left(b\left|I_{inj}\right|k^{2}t_{pw}^{k_{4}}{\left|Z_{\text{imag}}\right|}^{k_{6}}+1\right)\right],$$

where *V*_*elec*_ is the potential that builds up on the electrode–medium interface for an injected current *I*_*inj*_, pulse width *t*_*pw*_, and the imaginary part of the electrochemical impedance *Z*_*imag*_. The parameters *a, b, k*_2_*, k*_4_, and *k*_6_ are empirically determined parameters that depend on the electrode design, the experimental setup, the electrode material, the interface with the surrounding media and general variability in the injection process.^80^ The agreement of the model with experimental data in benchtop, as a function of the pulse width, injected current and impedance (Figure 6a–b). The electrochemical safety limit predicted by this model accounts for a greater set of electrode design parameters and is expressed as

2 $$\left|I_{\text{limit}}\right|={\left[\frac{\alpha D^{-d_{1}k_{6}}}{b{\left(t_{pw}\right)}^{k_{4}}}\left(e^{\frac{E_{mc}}{a}}-1\right)\right]}^{\frac{1}{k_{2}}},$$

where *d*_1_ captures the variance of the electrochemical impedance with the diameter of the electrode, and *E*_*mc*_ is the cathodal safety limit established from the CV measurement for hydrolysis. The *I*_*limit*_ predicted by the Shannon’s Equation can be expressed as

3 $$I_{\text{limit}\_\text{Shannon}}=\frac{D}{2t_{pw}}{\left(\pi 10^{\text{k}}\right)}^{0.5}.$$

The difference between the safety limits according to the Shannon’s Equation and this model shows the safety limits in our model are higher than with the Shannon’s Equation (Figure 6c). Equation 2 accounts for material and setup-based parameters that Shannon’s Equation does not take into consideration. To validate the applicability of Equation 2, the simulated data were verified for a held-out test data set consisting of measured *V*_*elec*_ from *in vivo* experiments in a pig’s cortex, using clinical depth, sEEG, and strip electrodes (Figure 6d–f).

Although typical electrochemical analyses of stimulation safety consider electrolysis as the leading cause of tissue damage and electrode failure, other mechanisms of tissue damage exist. Repeated, chronic stimulation below the electrolysis window has been known to cause damage to neurons,^84^ although the precise nature of this damage varies from case-to-case, and histological evaluation of the tissue damage is typically necessary for a more complete picture of the damage thresholds.

## Interconnect and packaging techniques

The approach of individually wiring each contact in clinical electrodes to an external circuit has persisted to this day, dating back to the clinical adoption of direct current stimulation in awake patients as instrumented by Penfield and described by Cushing.^85^ Today, touch-proof connectors are often used to connect neurostimulators or neural recording electronics to flexible silicone-embedded electrode arrays. These electrode arrays are robust, easily sterilized, and handled by neurosur-geons though they have poor compliance with the brain and have extremely poor spatial resolution and channel counts. Furthermore, they require technicians to plug in each channel into a port and keep a close record of the order in which each channel is plugged into the external electronics control system. For high-resolution systems with over tens of channels, this approach is clearly insufficient.

Thin-film fabrication and complementary metal oxide semiconductor (CMOS) fabrication techniques have long been capable of producing both rigid and flexible microelectrode arrays as well as rigid integrated circuits (ICs) with exceptionally small feature sizes; however, the integration of these two has remained a major challenge in the field. In the case of flexible polymer substrate-based electrode arrays, their marriage with rigid ICs is made difficult in part due to the often-high costs associated with CMOS post-processing at the water level, meaning that the fabrication must be done on small, diced samples, with increasing difficulty associated with introducing fabrication complexities. However, at a more fundamental level, mismatches in mechanical properties between rigid and flexible substrates lead to challenges in packaging, yield, and longevity of the implant. Furthermore, at a system level, other components beyond a single integrated circuit are typically required, making it difficult if not impossible to fabricate the entire system in a monolithic fashion. Thus, a common approach has been to fabricate the electrode array and the acquisition electronics separately and join them through conductive bonding processes or using high-density connectors.^33,86^ Alternative strategies gaining traction recently entail pushing analog front end (AFE) circuitry onto the electrode array substrate.^32,45^ Sharpened or thinned silicon substrates enable direct integration of multiplexing circuitry onto the same substrate as the electrode array, as in the case of the Neuropixels. However, this comes at the cost of restricting the materials and mechanical properties of the electrode arrays to that of single crystalline silicon.

By placing active electronics onto the electrode substrate, the burden placed on the insulation of said electronics is substantially elevated, and a combination of polymeric and ceramic films are typically required to achieve robust encap-sulation for chronic implants.^87–91^ This is still an active area of research, and there is an exciting opportunity for material and system-level advances to fill this need. One substantial need is for reliable, high-density, low impedance electrical feedthroughs through insulation layers. This is a challenge for several reasons, including delamination of metal/polymer or metal/ceramic layers, dissolution of ceramic materials, and fracture or pinhole defect propagation.

**Figure 7.**
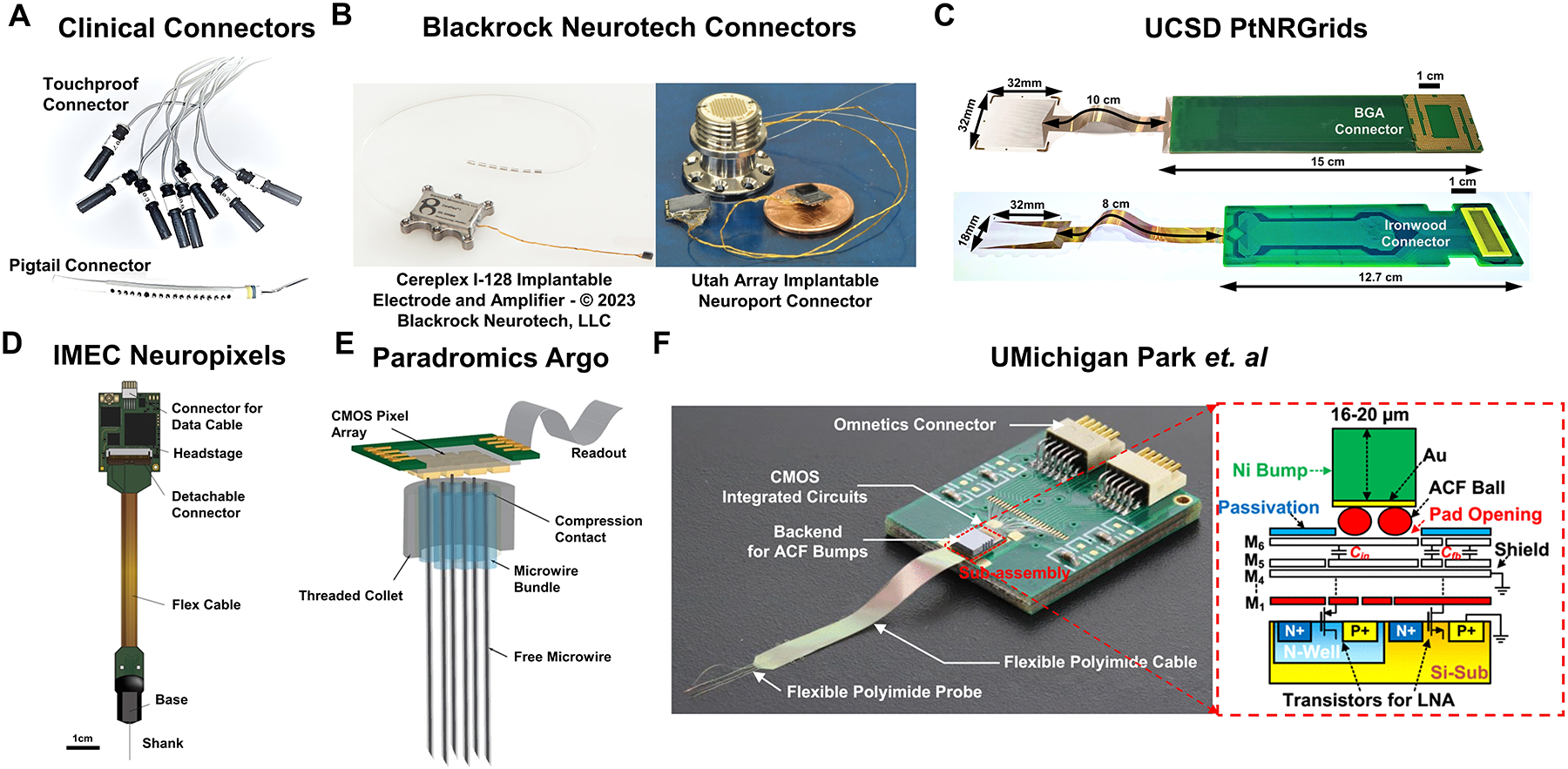
Connectorization to clinical and high channel count electrodes. (a) Example of commonly used connectors for current clinical electrodes. Each channel on these electrodes is individually routed to leads, which makes this approach unscalable for high channel counts. (b) Currently clinically adopted connector technologies developed by Blackrock Neurotech, for human implants using the Utah Microelectrode Arrays (Cereplex I-128 Implantable Electrode and Amplifier—© 2023 Blackrock Neurotech, LLC).^93^ (c) University of California, San Diego’s (UCSD) PtNRGrids use conventional land grid array (LGA)-type connectors and custom-built high-density Ironwood connectors to bond the flexible electrode to an extender printed circuit board to allow high-density connections to acquisition electronics. (d) IMEC Neuropixels provides 960 channels for recording and uses an integrated connector with a complementary metal oxide semiconductor (CMOS) digital neural probe integrated to the on-chip circuitry.^32^ (e) Paradromics Argo’s integrated connector vertically bonds interconnects on rigid substrates, allowing ultrahigh-density multi-thousand channel devices.^43^ (f) Flip-chip bonding technique developed at the University of Michigan–Ann Arbor using anisotropic conductive film (ACF) bonding technique to bond flexible substrates to CMOS chips, yielding a connection density of 167 channels/mm^2^.^92^ LNA, low-noise amplifier.

The decision to separate the electrode array from the acquisition electronics has historically also made the packaging and hermetic sealing of the active electronics more facile due to the physical separation of power supplies and the like from the passive recording contacts (and thus the biotic–abiotic interface). Current FDA-approved clinical systems often use the touch-proof connector-based Medusa adapter or the Blackrock Neuroport Connector used to interface with the Utah Microelectrode Arrays, both of which provide long, wired connections from the implanted electrode array to the recording electronics (Figure 7a–b). More recently, high channel count flexible parylene-C electrodes from UCSD were bonded to rigid extender PCBs using silver epoxy to separate the electrode from the recording interface, allowing for the sterilization of the assembled electrode arrays without the presence of active circuit elements, thus reducing the burden for packaging and sterile-sealing (Figure 7c).^33^ Although this approach successfully increased the channel count and simplified intraoperative translation, it still required routing each electrode contact to external acquisition electronics. This routing is the main limiting bottleneck in scaling to both higher channel counts and higher densities, with high-density electrode arrays requiring multiple metallization layers, adding parasitic capacitance, potentially compromising yield and increasing cost and time to fabricate.

To circumvent the 2D routing bottleneck, vertically bonded interconnects on rigid substrates enabled massive increases in density and channel counts (Figure 7d–e).^43^ This was achieved through gold microwire crimping directly onto the rigid CMOS substrate, and screws to hold the bonding in place. Although this approach has opened up new avenues for drastically increasing interconnect densities, the authors relied on stochastic methods coupled with microwire insulation thickness estimates to determine spatial maps rather than precisely aligning their microwire bundles to CMOS bonding pads. Another recently developed approach involved placing thick Ni bumps on a flexible polyimide electrode, and used an anisotropic conductive film (ACF) flip-chip bonding process to bond to a custom CMOS interface chip (Figure 7f), achieving a very high connector density of 167 channels/mm^2^.^92^

## Conclusion and future directions

Recent advances in electrode fabrication technology and the realization of microelectrode arrays with low impedance have enabled the development of high-density, high channel count electrode arrays, improving our ability to record and modulate neuronal activity. In this article, we focused on the impact of the electrode materials and fabrication meth-odologies for recording and stimulation performance. The long-term stability, the mechanical properties, data processing, and biocompatibility infringe other important material considerations for electrode design and translation that were not discussed here.

In addition to the electrode interface with tissue, intercom-nections between the contacts on the electrode and the acquisition electronics pose additional technological challenges. The intersection between flexible electrode arrays and integrated circuits is inevitable for compact connectorization and for high channel count systems. For stimulation and active electrode technologies in particular, insulation is a major concern, where leakage and failure paths can lead to catastrophic failure of the implant and potential harm to the subjects. Thus, there exists an exciting opportunity for novel materials and packaging techniques to fill the stringent needs of providing hermetic and robust biocompatible insulation in dense form-factors.

**Figure 8.**
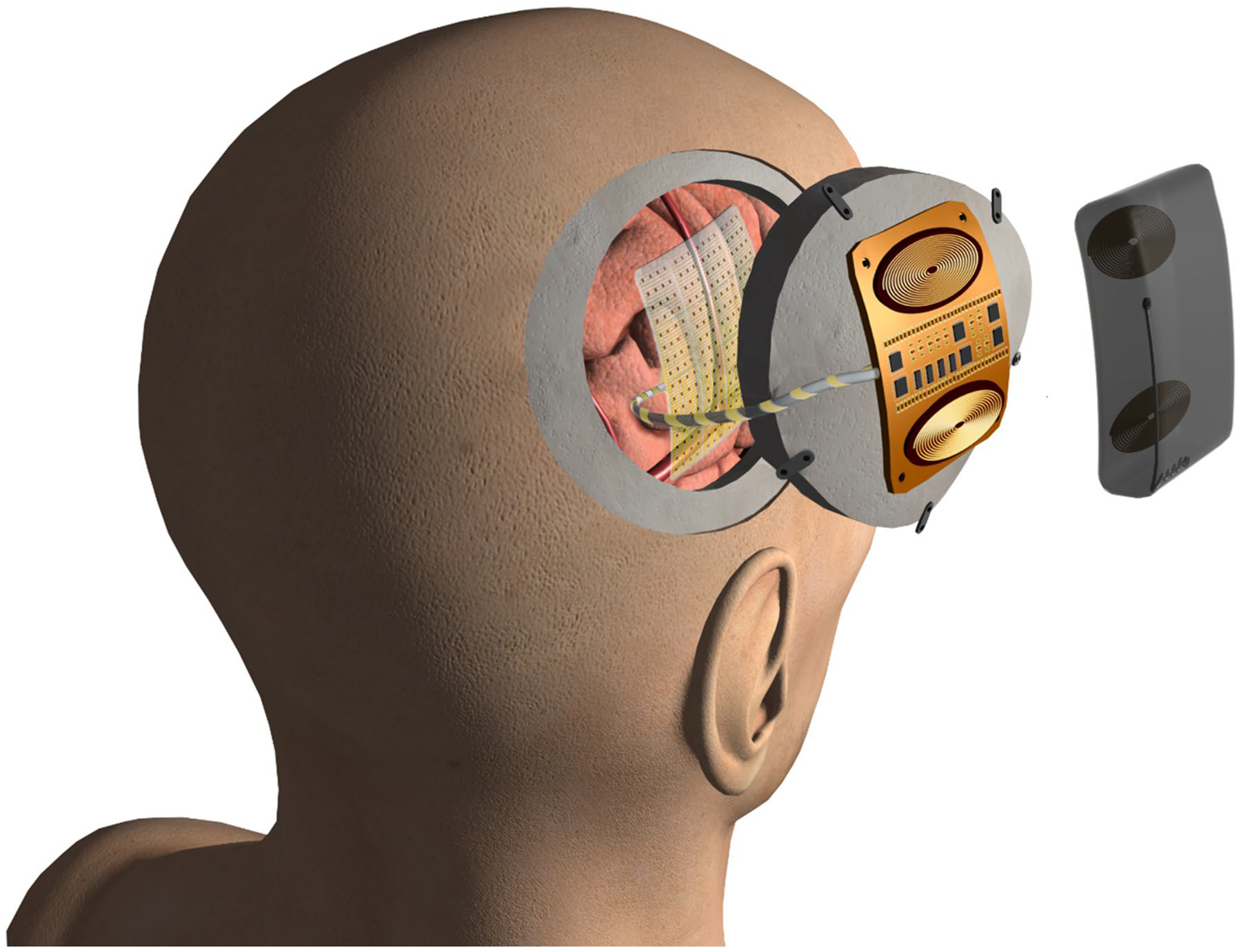
Schematic of a human-grade high-density, high channel count electrode array with a wireless acquisition system with simultaneous recording and stimulation capabilities for chronic implants, currently under development by the present authors and collaborators.

As major advances are made in almost every aspect of electrode array technology, several new therapeutic and diagnostic avenues will open for the adaptation of these devices. To facilitate the adoption of these devices for chronic implants in non-clinical environments, there is a need to develop complementary systems that facilitate wireless powering and untethered data transmission. This will reduce the risks of infection of the usually externalized wires, simplify the mentoring procedure and needed equipment to facilitate one-date ambulatory monitoring outside the hospital environment. Therefore, there has been a recent push toward developing wireless devices that can simultaneously record and stimulate neural activity, for therapeutic applications for Parkinson’s disease^94^ and epilepsy. This necessitates the consideration of electrical power and heat management to ensure patient safety, at sufficient data bandwidths and power requirements for managing the implants add necessarily important technical considerations and challenges that must be overcome. An artistic illustration of how such a system could be implanted and a coarse view of its elements are shown in Figure 8, which depicts a multi-thousand channel wireless μECoG system that the present authors and collaborators are developing.

## Figures and Tables

**Table I.** An overview of the state-of-the-art electrode arrays, comparing the channel count, dimension, substrate material, contact material, test subjects, and target brain regions for application.

Group	Key Device Figure	Channel Count	Dimension	Substrate Material	Electrode Contact Material	Subject	Target Brain Region
UCSD PtNRGrids^33^	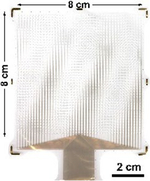	1024–2048	Total length up to 28 cm, Recording area from 25 mm^2^ to 6400 mm^2^, 6.6 μm thickness	Parylene C	Pt Nanorods (30 μm)	Rodent, Pig, NHP, Human	Primary Somatosensory Cortex (Motor, Sensory, Barrel)
UCSD Depth Electrodes^58^	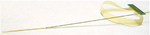	128	Total length up to 28 cm, Recording length ranges from 1.89 to 7.65 mm, 15 μm thickness	Polyimide	Pt Nanorods (30 μm), PEDOTPSS (20 μm)	Rodent, Pig, NHP, Human	Primary Somatosensory Cortex, Deep Brain
Duke University LCP TF Surface Electrodes^59^	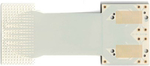	61–256	Recording area from 3.4 × 3.4 mm^2^ to 38 × 21 mm^2^, 50 μm thickness	Liquid-Crystal Polymer	Ptlr (200 μm, micro diameter to 2.3 mm, macro diameter)	Rodent, NHP, Human	Primary Motor Cortex, Auditory Cortex (pSTG)
UCSF+LLNL Depth Electrode^60^	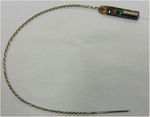	32	Total length: 19 mm, axial spacing 1 mm edge-to-edge, 0.301 mm edge-to-edge circumferentially	Polyimide	Ptlr (1.5 mm × 0.5 mm)	Human (epilepsy patients)	Temporal Lobe
UCSF+LLNL Surface Electrode^60^	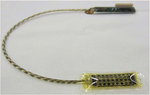	32	Recording area of 15.2 × 7.2 mm^2^, ~0.8 mm thickness	Polyimide, encased in over-molded in silicone	Ptlr (1.2 mm diameter)	Human (epilepsy patients)	Temporal Lobe
IMEC Neuropixels^32^	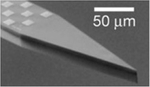	960 electrodes, with simultaneous recording capability from 384	10 mm long non-tapered shank with 70 μm × 20 μm cross-section	CMOS	TiN (12 μm × 12 μm squares)	Rodents, NHP, Humans	Intracortical: (Motor, Visual, Thalamus, Hippocampus, Striatum)
Neuralink Threads^61^	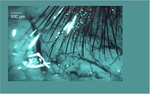	1536 to 3076 (32 ch per thread)	Up to 20 mm length, 5 to 50 μm width, 4 to 6 μm thickness; 96 threads over 4 mm × 7 mm area	Polyimide with Parylene C	IrOx (14 μm × 24 μm)	Rodent, Pig, NHP	Intracortical: Motor Cortex
Osaka University Surface Electrode^51^	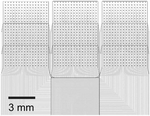	1152	Recording area of 14 × 7 mm^2^, over 20 μm thickness	Parylene C	Pt Black (50 μm × 50 μm)	NHP	Primary Somatosensory Cortex
University of Freiberg, Germany^62^	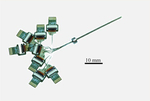	64–128	22 mm deep implantation, 0.8 mm diameter	Polyimide, Silicone inside inner diameter	Pt (35 μm diameter)	Rodent, Monkey	Parietal Cortex
The Argo, Paradromics^36^	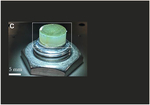	65,536 (*in vivo* recording from 100 to 1300 in rodent, 30,000 in sheep)	1 mm length microwire, 18 μm diameter	Ptlr Core Microwire with Alumina and Parylene C insulation	PtIR core	Rodent, Sheep	Intracortical: Primary Somatosensory Cortex
Nanoelectronic thread (NET) electrodes, Rice University^63^	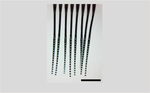	2304 (18128 channel modules)	8 shanks, 16 contacts, 60 μm electrode pitch, 10 μm × 1.5 μm)	SU-8 micro-trenches + Tungsten microwires (25 μm diameter) as shuttles	PEDOTPSS (15 μm × 15 μm to 25 μm × 25 μm)	Rodent	Intracortical: Primary Sensory Neocortex (Visual, barrel, motor)
